# Test–Retest Reproducibility of the Microperimeter MP3 With Fundus Image Tracking in Healthy Subjects and Patients With Macular Disease

**DOI:** 10.1167/tvst.7.1.17

**Published:** 2018-02-07

**Authors:** Stefan Palkovits, Nino Hirnschall, Stefan Georgiev, Christoph Leisser, Oliver Findl

**Affiliations:** 1 VIROS–Vienna Institute for Research in Ocular Surgery, A Karl Landsteiner Institute, Hanusch Hospital, Vienna, Austria; 2 Moorfields Eye Hospital NHS Foundation Trust, London, United Kingdom

**Keywords:** microperimetry, MP3, visual field, macula disease

## Abstract

**Purpose:**

To evaluate the test–retest reproducibility of a novel microperimeter with fundus image tracking (MP3, Nidek Co, Japan) in healthy subjects and patients with macular disease.

**Methods:**

Ten healthy subjects and 20 patients suffering from range of macular diseases were included. After training measurements, two additional microperimetry measurements were scheduled. Test–retest reproducibility was assessed for mean retinal sensitivity, pointwise sensitivity, and deep scotoma size using the coefficient of repeatability and Bland-Altman diagrams. In addition, in a subgroup of patients microperimetry was compared with conventional perimetry.

**Results:**

Average differences in mean retinal sensitivity between the two study measurements were 0.26 ± 1.7 dB (median 0 dB; interquartile range [IQR] −1 to 1) for the healthy and 0.36 ± 2.5 dB (median 0 dB; IQR −1 to 2) for the macular patient group. Coefficients of repeatability for mean retinal sensitivity and pointwise retinal sensitivity were 1.2 and 3.3 dB for the healthy subjects and 1.6 and 5.0 dB for the macular disease patients, respectively. Absolute agreement in deep scotoma size between both study days was found in 79.9% of the test loci.

**Conclusion:**

The microperimeter MP3 shows an adequate test–retest reproducibility for mean retinal sensitivity, pointwise retinal sensitivity, and deep scotoma size in healthy subjects and patients suffering from macular disease. Furthermore, reproducibility of microperimetry is higher than conventional perimetry.

**Translational Relevance:**

Reproducibility is an important measure for each diagnostic device. Especially in a clinical setting high reproducibility set the basis to achieve reliable results using the specific device. Therefore, assessment of the reproducibility is of eminent importance to interpret the findings of future studies.

## Introduction

Diseases of the macula affect visual function and lead to significant alteration in everyday life. Among others, age-related macular degeneration is a significant cause of visual impairment as mainly central vision is impaired.^1,2^ In daily routine, visual acuity as a functional parameter is used to monitor macular disease. Nevertheless, for many tasks such as reading, fixation stability and paracentral retinal sensitivity are important factors.^3,4^ Assessment of paracentral retinal sensitivity using conventional perimetry is, however, challenging as patients with macular disease often show unstable fixation as a result of central scotomas. In contrast to conventional perimetry, microperimetry utilizes fundus imaging and motion tracking to ensure precise stimulation of a certain location of the retina. This so-called fundus-related perimetry relates retinal sensitivity testing with morphology and is therefore a powerful tool in evaluating macular disease.

Recently, a new microperimeter with improved fundus image tracking (MP3, Nidek Co, Japan) became available. The system comprises a nonmydriatic fundus camera with 45° field of view. During the measurement, an infrared image is used for motion capturing. In contrast to previous devices, the tracking frame rate is 30 Hz, where proper fixation and the correct position of the stimulation grid are evaluated 30 times per second.

Assessing retinal sensitivity using microperimetry has become a highly studied method in recent years. Microperimeter 1 (MP1), the former version of the MP3, was used in several clinical trials. In these studies, the device showed good repeatability^5–7^ and a normal value database has been established.^7^ Retinal sensitivity of patients with age-related macular degeneration,^8,9^ retinal vein occlusion,^10^ birdshot chorioretinopathy,^11^ cystoid macular edema,^12^ or epiretinal membrane^13^ was investigated using the MP1. Other studies indicate that microperimetry might be a valuable method in visual rehabilitation.^14,15^ All mentioned studies indicate that microperimetry is a promising technique in evaluation of central retinal sensitivity.

Previous studies compared microperimetry with static perimetry.^16,17^ In comparison with conventional perimetry, microperimetry comes with several advantages, such as motion tracking and fundus related perimetry.

The present study was designed to evaluate the test–retest reproducibility of retinal sensitivity assessment using the MP3 in healthy subjects and patients with macular disease. In this respect, mean retinal sensitivity, pointwise retinal sensitivity, and deep scotoma size were evaluated. Further in a subgroup of macula patients, reproducibility and deep scotoma size were compared between microperimetry and conventional perimetry.

## Methods

Ten healthy subjects and 20 patients with macular disease were included in this study. Prior to study entry, a screening examination, with assessment of best corrected distance visual acuity, slit lamp biomicroscopy, fundoscopy, and optical coherence tomography (OCT; Spectralis, Heidelberg Engineering, Heidelberg, Germany) was performed. Men and women aged 18 years or older were recruited in the outpatients' department of the Department of Ophthalmology at the Hanusch Hospital, Vienna. To be included into the healthy subjects group, absence of any ocular pathology or any history of ophthalmological disease was mandatory. In the macular disease patient group, a significant macular disease such as geographic atrophy, drusen maculopathy, or epiretinal membrane had to be present. Patients with exudative age-related macular degeneration, cystoid macular edema, or diabetic macula edema were excluded from the study due to the possibility of short-term fluctuations of macular function within the study period. Presence of any kind of opacity of the ocular media which could have had interfered with the study relevant procedures excluded the respective subject from the study.

All study related documents were reviewed by the ethics committee of the city of Vienna and written informed consent was obtained in all subjects prior to study entry. The study procedures followed the guidelines of Good Clinical Practice and adhered to the Tenets of the Declaration of Helsinki.

After the study enrollment, three measurements with the MP3 were scheduled on three different days within 1 week. As recommended by Wong et al.^6^ the baseline examination (test 1) was classified as the training measurement.

### Microperimetry Testing Protocol

Microperimetry assessments were conducted in a dark room (ambient illumination < 1 lux) while the contralateral eye was patched. The examinations were performed by two experienced readers (S.P. and S.G.). The stimulation pattern consisted of 56 test locations arranged in a 2° by 2° grid centered at the fovea. The standard 1° red circle was set as the fixation target and the stimulation algorithm followed the 4-2-1 staircase. The size of the stimulus was Goldmann III (4 mm^2^, 25.7 arc minutes), and its color and duration were set to white and 100 ms, respectively. The starting threshold of the baseline measurement was set to 17 dB for one test location in each quadrant. The final results of these first measurements served as the starting threshold of the remaining points in the respective quadrant. During the second (test 2) and the third (test 3) microperimetry assessments the follow-up function was used, whereby the starting threshold value of each point was automatically set in correspondence to the final result of test 1. For follow-up examination a reference infra-red image of the actual examination was automatically compared with a reference image of the baseline test and the build in software created an overlay of both imaging to get the exact same position of the stimulation grid. If the automatic alignment failed or resulted in incorrect positioning, the examiner chose two characteristic landmarks (e.g., prominent vessels or vessel bifurcations) of the fundus for manual alignment.

In case of significant eye movements, the test was paused automatically. Furthermore, the test was paused, if the patient required a break. After proper realignment of the study eye the examination was continued. At the end of the examination, a fundus photography was taken, which again was overlaid with the infra-red image to assure proper alignment.

Conventional perimetry (Octopus 101, Haag-Streit AG, Germany) was performed in addition to microperimetry on a subgroup of 15 subjects in the macular patients group. Thereby, exactly the same stimulation grid (56 test locations, 2° × 2° grid) and stimulation settings (Goldmann III, white stimulus, 100 ms stimulation duration, 4-2-1 staircase) were applied. Maximum stimulation intensity of the Octopus 101 is 1910 cd/m^2^.

### Statistical Analyses

Data following a normal distribution are presented using means and standard deviations, otherwise median and interquartile range (IQR) are presented. The Shapiro-Wilk test for normality was used to assess the distribution of the outcome parameters. For mean retinal sensitivity, all 56 points of each of the three examinations were averaged. For pointwise retinal sensitivity, each test location was considered separately. Retinal sensitivity data for all three measurements (test 1, test 2, test 3) are presented, and differences between each test were calculated using Friedman's 2-way analysis of variance (ANOVA) and Wilcoxon signed rank test. We assessed reproducibility between test 2 and test 3. As such, differences in mean retinal sensitivity as well as pointwise sensitivity were computed. Deep scotoma size was defined as the number of test loci showing a retinal sensitivity of 0 dB. The change in this value was calculated between test 2 and test 3. Bland-Altman plots and the coefficient of repeatability were utilized to evaluate all three parameters' reproducibility. Coefficient of repeatability calculates as two times the standard deviation of the difference between both tests.

Mean retinal sensitivity as well as deep scotoma size were evaluated and compared between microperimetry and conventional perimetry. Comparison in deep scotoma size between microperimetry and conventional perimetry was performed using Mann-Whitney *U* test.

## Results

Ten healthy subjects (5 males, 5 females) were recruited for the healthy study group (median age, 31; IQR 23–34). Twenty patients (12 males, 8 females) were recruited for the macular disease group (median age, 77.5; IQR 74–83). Median distance corrected visual acuity in the healthy subjects group and the macular disease patient group were −0.1 logMAR (IQR 0 to −0.18) and 0.8 logMAR (IQR 1.02–0.6), respectively. Within the macular patients group, eight patients presented with geographic atrophy, three patients had an epiretinal membrane, seven patients had a drusen maculopathy, one patient suffered from central serous chorioretinopathy, and one patient from a retinal pigment epithelium tear. Fixation stability was graded with the build in software as stable fixation when 75% or more of the fixation points fall inside a 2-degree circle around the point of fixation, as relative stable when 75% or more of the fixation points fall inside a 4-degree circle (but less than 75% are inside the 2-degree circle) and as unstable when less than 75% of the points fall inside the 4-degree circle. All subjects in the healthy study group showed a stable fixation. In the macula group, fixation stability was graded as stable in 11 subjects, as relatively stable in six and as unstable in three patients during test 2. Comparable results were achieved during test 3 (12 stable, 5 relatively stable, 3 unstable). All subjects completed all measurements and MP3 data of all assessments were considered for the final statistical evaluation.

### Reproducibility of Microperimetry

**Figure 1:**
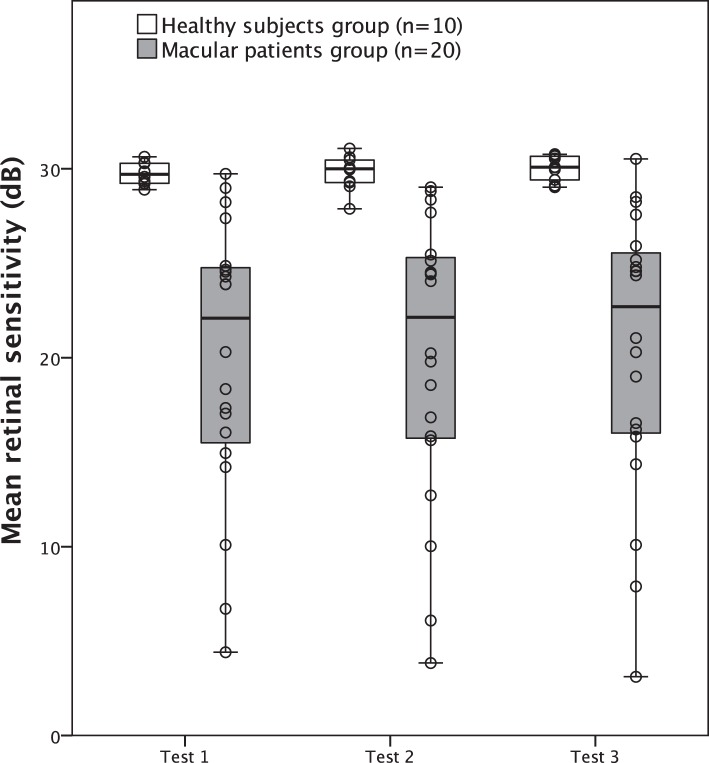
Boxplots with overlaid scatterplot of mean retinal sensitivity for both study groups (boxes indicate IQR; whiskers indicate range).

Mean retinal sensitivity for the healthy group of test 1, test 2, and test 3 were 29.7 ± 0.6 dB (median, 29.7 dB; IQR 29.2–30.3), 29.8 ± 0.9 dB (median, 30 dB; IQR 29.2–30.5) and 30.0 ± 0.7dB (median, 30.1 dB; IQR 29.3–30.7), respectively. The corresponding values for the macular disease patients group were 20.0 ± 7.3 dB (median, 22.1 dB; IQR 15.2–24.8), 20.1 ± 7.5 dB (median, 22.1 dB; IQR 15.7–25.4), and 20.4 ± 7.5 dB (median, 22.7 dB; IQR 15.9–25.7). No significant difference in mean retinal sensitivity was found for the healthy subjects and macular patients (*P* = 0.202 and *P* = 0.101, respectively, Friedman's 2-way ANOVA). Average differences in pointwise retinal sensitivity between test 2 and test 3 were 0.26 ± 1.7 dB (median, 0 dB; IQR −1 to 1) for the healthy and 0.36 ± 2.5 dB (median, 0 dB; IQR −1 to 2) for the macular patients group. Wilcoxon signed rank test revealed a significant difference to 0 for both groups (*P* < 0.001). In contrast, average differences between test 1 and test 2 were smaller (healthy subjects: 0.06 ± 1.7 dB; macular patients: 0.08 ± 3.89 dB) and no significant difference could be found (*P* = 0.11 and *P* = 0.81, respectively). Boxplots for retinal sensitivity are shown in Figure 1.

**Figure 2:**
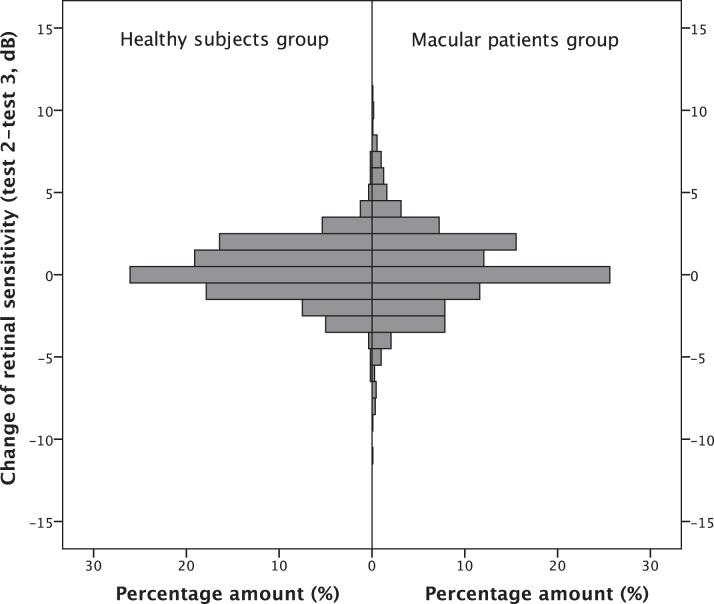
Histogram of percentage amount of change in pointwise sensitivity between test 2 and test 3. Left panel, data for healthy subjects; right panel, data for macular patients group.

In total 560 test loci in the healthy study group and 1120 test loci in the macula patients group were assessed during each microperimetry test. The measured sensitivity values in test 2 and test 3 were the same in 146 (26%) and 287 (26%) test loci in the healthy and the macular patients group, respectively. Four hundred eighty-seven (87%) test locations in the healthy group and in 814 (73%) of the macular patients group were within a range of −2 to 2 dB. Summary of the change in each test location is shown in Table 1 and Figure 2.

**Table 1:**
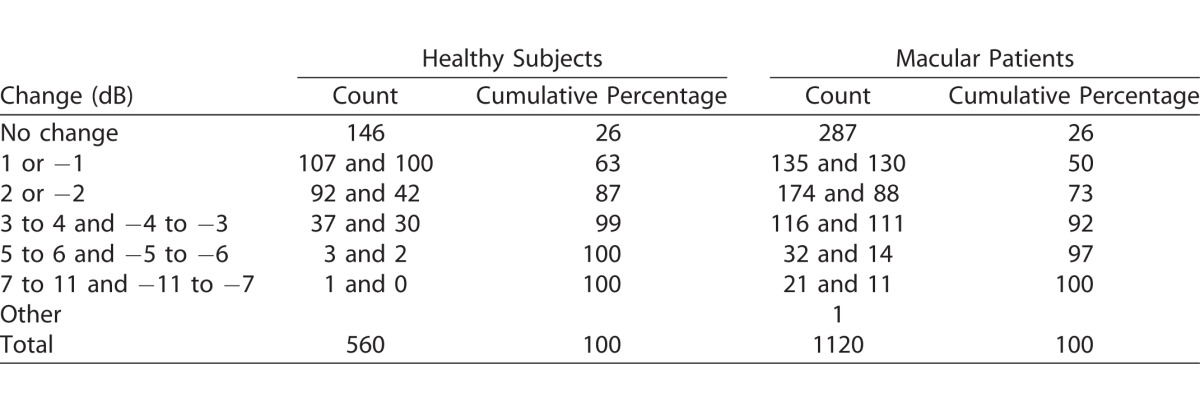
Frequency of Change in Pointwise Retinal Sensitivity for Healthy Subjects and Macula Patients Between Test 2 and Test 3

**Figure 3:**
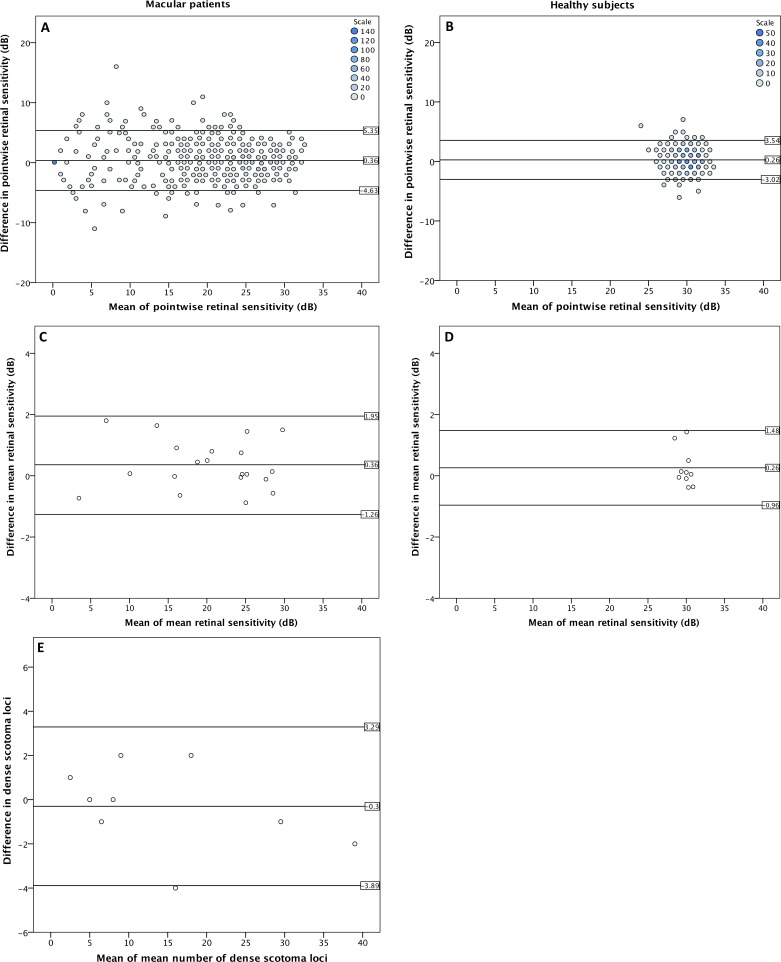
Bland-Altman plots; (A, B) Binned Bland-Altman plots for pointwise retinal sensitivity (presenting all data points of the measurements) for macula patients and healthy subjects, respectively. (C, D) Bland-Altman plots for mean retinal sensitivity (presenting the average of all data points of the measurements) for macula patients and healthy subjects, respectively. (E) Bland-Altman plots for deep scotoma size for 10 macula patients (at least one test location with a retinal sensitivity of 0 dB). Horizontal lines and numbers indicate 95% upper limit, the mean, and the 95% lower limit of the difference.

Figures 3A–3D illustrate the Bland-Altman plots for mean and pointwise retinal sensitivity for both study groups.

Coefficients of repeatability were calculated between test 2 and test 3. Coefficients of repeatability for the mean retinal sensitivity were 1.2 dB for healthy subjects and 1.6 dB for macula patients. The coefficients of repeatability for the pointwise retinal sensitivity were 3.3 dB for the healthy subjects and 5.0 dB for the macula patients.

Bland-Altman plot for pointwise retinal sensitivity for the macular patients group showed a significant floor effect of the results. No significant ceiling effect was observed in both study groups. After correction of the floor effect coefficient of repeatability in the macula patients group was 5.3 dB.

**Figure 4:**
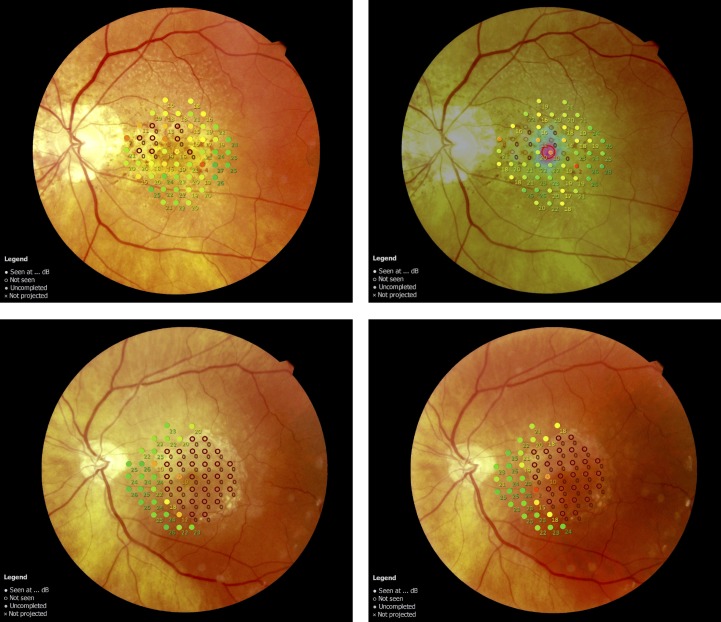
Microperimetry finding of two patients with geographic atrophy. Findings from test 2 are presented on the left side, those from test 3 on the right. First patient (top row) presented a small area of geographic atrophy, with perfect agreement between test 2 and test 3. A larger area of geographic atrophy was found in the second patient (bottom row). Correspondence of deep scotoma size differed in one test location in this patient.

Ten subjects in the macular patients group showed at least one test location with retinal sensitivity of 0 dB in test 2 or test 3. Figure 4 illustrates two patients with deep scotoma. Absolute agreement in deep scotoma size between test 2 and test 3 was found in 79.9% of the test loci. The median number of test loci with measurements of 0 dB were 9 (IQR 5.75–21.5) and 8 (IQR 6.5–21) for tests 2 and 3, respectively. Median change of deep scotoma size was 0 (IQR −1.25 to 1.25; *P* = 0.732, Wilcoxon signed rank test). Three subjects showed absolute agreement in deep scotoma size and six subjects differed in one or two test locations. One subject had a difference of four locations. The Bland Altman plot for deep scotoma size is shown in Figure 3E.

### Comparison Between Microperimetry and Conventional Perimetry

**Figure 5:**
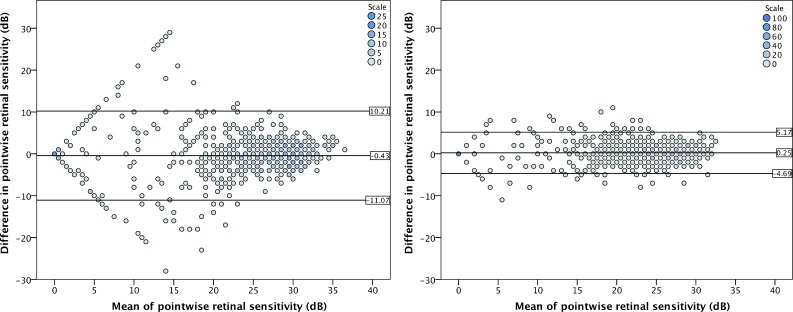
Binned Bland-Altman plots for conventional perimetry (left side) and microperimetry (right side) in a subgroup of macula patients (15 subjects; 860 test locations). Horizontal lines and numbers indicate 95% upper limit, the mean, and the 95% lower limit of the difference.

In addition to microperimetry, conventional perimetry was performed in 15 subjects of the macular patients group (total 840 test loci during each test). The coefficient of repeatability for pointwise retinal sensitivity using microperimetry in this subgroup was 4.9 dB, and 10.6 dB using conventional perimetry. Figure 5 illustrated Bland-Altman plots for both devices between test 2 and test 3.

Eight out of 15 patients had at least one deep scotoma locus in microperimetry in both tests. In contrast, six patients had deep scotoma during test 2 and seven during test 3 in conventional perimetry. Median difference in deep scotoma size between microperimetry and conventional perimetry was −4.5 (IQR −8.25 to −3.0) for test 2 and −5.5 (IQR −10.0 to −1.25) for test 3. Though median variation of deep scotoma size between test 2 and test 3 was similar between both devices (*P* = 0.328, Mann-Whitney *U* test), variation of scotoma location was larger in conventional perimetry. Hence, full agreement of deep scotoma between test 2 and test 3 in microperimetry was reached in 94.5% of all test points with a retinal sensitivity of 0 dB, whereas the value was 41.2% in conventional perimetry. Median number of deep scotoma loci in microperimetry was 9 (IQR 5.75–21.5) for test 2 and 8 (IQR 6.5–21.0) for test 3. In conventional perimetry, median number of deep scotoma was 2 (IQR 0.25–10.75) for test 2 and 4 (IQR 1.0–6.5) for test 3. Using microperimetry, three patients showed absolute agreement in deep scotoma size, four patients differed in one or two loci and one subject differed in four loci. In conventional perimetry, only one subject reached absolute agreement, five subjects differed in five or less loci, and one subject differed in eight test loci.

## Discussion

The present study evaluated the reproducibility of the MP3 in healthy subjects and in patients with macular disease. Test–retest reproducibility of three parameters was analyzed: mean retinal sensitivity, pointwise sensitivity, and deep scotoma size. All three parameters showed adequate reproducibility in both groups. Reproducibility of microperimetry was higher as compared to conventional perimetry in the subgroup tested with both devices.

The measurement of retinal sensitivity using the MP3 was well tolerated in every subject under study and is therefore an applicable technique in healthy subjects as well as in elderly patients with macular disease. This acceptance is further enhanced by the fact that the patient or the examiner can pause the examination at any point in time to prevent significant fatigue effects.

Former studies identified a significant learning effect in the microperimetry using the MP1, indicated by an increase in retinal sensitivity between the first and the second measurement.^6,18^ Similar effects have been shown for conventional perimetry.^19,20^ However, we observed no significant difference in mean retinal sensitivity in the healthy study group as well as in the macular patient group. The average difference between test 2 and test 3, however, was slightly larger as compared to the average difference between test 1 and test 2. In that respect, the former was significantly different to zero. Nevertheless, the difference was very small and can be considered as not clinically relevant. As such, our results indicate that there is only a small if any learning effect in our study population using the MP3. In that respect one has to consider that the MP3 utilizes an improved motion tracking system and full automatic measurement procedure, which appear to reduce the bias of the MP3 examination. However, further studies are needed to investigate the influence of patient's experience using the device on its readings.

To the best of our knowledge, this is the first study investigating reproducibility of the MP3 in a clinical setting. A comprehensive evaluation of the test–retest variability in patients with macular disease for the MP1 was conducted by Chen et al.^5^ In detail, they evaluated 68 test locations in the central 20° of the posterior pole, similar to the grid in our study. However, a different test algorithm (4-2 staircase) and a longer stimulation duration (200 ms) were used by Chen et al.^5^ Their findings showed a good repeatability of this method. Coefficient of repeatability for mean retinal sensitivity was 1.81 dB and for the pointwise retinal sensitivity between 3.5 and 7.2 dB. These findings are in good agreement with the data of the present study (1.6 and 5.0 dB, respectively). Nevertheless, one has to consider the slightly different testing protocols. The test algorithm utilized by Chen et al.^5^ (4-2 staircase) has a lower resolution of retinal sensitivity values as the algorithm used in the present trial (4-2-1 staircase), which might have a decreased chance to detect slight variability between the measurements in the previous study. This fact is further underlined by the finding that about 26% of the test locations show no change between both measurements in our study (Chen et al.^5^: 47%). Agreement for values between −2 and 2 dB was also slightly different (MP1: 81% vs. MP3: 73%). This difference might be explained by the different test algorithm and by the composition of the patients' population.

The MP1 has a narrower dynamic range of stimulus luminance when compared to the MP3 (MP1: 0 to 20 dB vs. MP3: 0 to 34 dB), which has led to significant floor and ceiling effects in the former MP1 study.^5^ Like the mentioned study, a floor effect was also observed in our trial. Due to the higher dynamic range of the MP3, only a slight ceiling effect might be present in the present study, as only a very few subjects reached the highest retinal sensitivity. Nevertheless, the effect on reproducibility is assumed to be very small. Further, Bland-Altman plot show that the variance of retinal sensitivity values in the macular patients group were higher at the middle and bottom retinal sensitivity values, which is common in perimetry examinations. One has to consider that a microperimetry test within this retinal sensitivity range might have lower reproducibility values.

Maximum stimulation luminance of the MP3 is 3193 cd/m^2^ (0 dB), and the minimum luminance is 11 cd/m^2^ (34 dB). Background illumination is constant at 10 cd/m^2^. The maximum and minimum luminance levels for the MP1 are 129 cd/m^2^ (0 dB) and 3 cd/m^2^ (20 dB). The background illumination of the MP1 is 4 dB. Thus, it does not come as a surprise that the MP3 can test at a lower light intensity threshold, as the stimulation contrast can reach much lower levels (MP3: 35.4:31.4; MP1: 8:4). Therefore, the MP3 exceeds the sensitivity of a healthy retina in most cases. On the other hand, due to the higher maximum stimulation intensity scotoma detection is more accurate. Our data reflect this fact as only 14 out of 3360 (total amount of test locations on both study days) test locations, all in the healthy group, showed the highest possible sensitivity (34 dB), whereas 283 out of 3360 test locations, all in the macular disease patient group, had a value of 0 dB. As depicted in Figure 3A, values below mean pointwise retinal sensitivity of 3 dB in the macular patients group have obviously, a lower variance as compared to the rest of the measurements. After excluding these values, the coefficient of repeatability for the macular patients group was slightly higher (without correction: 5.0 dB; with correction 5.3 dB). Nevertheless, our data suggest adequate repeatability in macular patients.

Even though a large proportion of patients in the macular disease group showed at least one location of absolute scotoma (10 out of 20), reproducibility in this group was still very good. Nine patients tended to have a reduced fixation ability during test 2 (six relative stable; three unstable). During test 3, eight patients showed reduced fixation ability (five relative stable; three unstable). Despite poor fixation, assessment of retinal sensitivity could be performed accurately, and deep scotoma size was comparable between test 2 and test 3 (*P* = 0.732, Wilcoxon signed rank test). Figure 4 illustrates both microperimetry assessments for two example patients with geographic atrophy. One patient had a high difference in number of deep scotoma test loci (test 2: 14; test 3: 18; see Fig. 3E). Whether this difference is due to the large area of geographic atrophy or due to a reduced fixation ability is not clear. Nevertheless, our data indicate that the MP3 is a valuable tool in assessing the spread of atrophic areas on the retina and its progression over time.

Small sample size and diversity of pathologies in the macula patients group are limitations of the present study. In the strictest sense, the results of the present study only refer to the diseases investigated. Nevertheless, the findings show high reproducibility in patients with impaired retinal function in general. The pathologies investigated in the study were stable within the study period, evaluated by OCT. Therefore, influence of disease progression can be excluded. Currently, no normal value database, which has been introduced for the MP1,^7^ is available for the MP3. For that reason, it is currently not possible to compare our results with an age-matched healthy control collective.

Reproducibility of microperimetry was superior to conventional perimetry in our study population, and the former showed a lower coefficient of repeatability (4.9 dB vs. 10.64 dB). The implementation of the follow-up function, motion tracking, and precise stimulation location might have led to more repeatable results. Furthermore, microperimetry was more sensitive in detecting deep scotoma. The reproducibility in deep scotoma detection was also higher with microperimetry. However, the field of view in microperimetry is much smaller as compared to conventional perimetry, limiting microperimetry to the evaluation of the posterior pole. Therefore, its application in other ocular pathologies like glaucoma needs to be addressed in further studies.

## Conclusion

This is the first study investigating the reproducibility of the Nidek's microperimeter MP3. Our findings indicate an adequate test–retest reproducibility for mean retinal sensitivity, pointwise retinal sensitivity, and deep scotoma size in healthy subjects and patients with a range of macula diseases. Microperimetry can be an important jigsaw piece in the broad spectrum of multimodal retinal imaging techniques in the clinical assessment of patients with macula disease. Furthermore, microperimetry seems to be more reproducible to conventional perimetry in assessing central retinal sensitivity in this group of patients. Highly reproducible assessment of retinal sensitivity, which has been shown in this study, is a prerequisite in the evaluation of central macula function and in assessing disease progression. MP3 microperimetry has a higher range of stimulation intensities, compared to the MP1, and therefore only a minimal ceiling effect. Further studies are needed to establish a normal-value database for the MP3 and to set cutoffs in the evaluation of progression in the respective macula pathology.
